# Retraction: Combination of mTOR and EGFR kinase inhibitors blocks mTORC1 and mTORC2 kinase activity and suppresses the progression of Colorectal Carcinoma

**DOI:** 10.1371/journal.pone.0330746

**Published:** 2025-08-26

**Authors:** The PLOS One Editors

After this article [1] was published, concerns were raised regarding results presented in Figs 1–3.

Specifically,

- There appear to be full or partial overlaps in the following panels in this article [1], despite the panels representing different experimental conditions:
  - Fig 1C Colo320 mTOR lanes 1–4 and Fig 1C HT29 mTOR lanes 2-5
  - Fig 2B DLD-1 p-AKT right panel, Fig 2B HT29 p-AKT right panel, and Fig 2C HT29 p-AKT right panel
  - Fig 2B DLD-1 AKT left panel lanes 1-5 and Fig 2B DLD-1 AKT middle panel lanes 2-6
  - Fig 2B DLD-1 β-actin left panel and Fig 2B DLD-1 β-actin right panel
  - Fig 2B HT29 AKT left panel lanes 3-6, Fig 2B HT29 AKT middle panel lanes 2-5, and Fig 2B HT29 AKT right panel lanes 1-4
  - Fig 2B DLD-1 p-EGFR right panel and Fig 3A p-AKT left panel
  - Fig 2B HT29 p-AKT middle panel and Fig 3A p-S6 left hand panel
- The following panels presented in this article [1] appear similar to panels published in [2] despite representing different experimental conditions:
  - Fig 1C DLD-1 p-AKT in [1] and Fig 1D Mia-Paca2 p-Akt (Ser473) in [2]
  - Fig 1C HT29 AKT in [1] and Fig 1D CFPAC-1 Akt in [2]
  - Fig 1C HT29 p-S6 in [1], Fig 1C DLD-1 p-S6 in [1], and Fig 1D Capan-1 p-S6 (Ser235/236) in [2]
  - Fig 1C Colo320 p-4E-BP1 in [1], Fig 1C HT29 p-4E-BP1 in [1], Fig 1C SW948 p-4E-BP1 in [1], and Fig 1D PANC-1 p-4E-BP1 (Ser37/46) in [2]
  - Fig 1C DLD-1 p-4E-BP1 in [1] and Fig1D Capan-1 p-4E-BP1 (Ser37/46) in [2]
  - Fig 1C Colo320 4E-BP1 in [1] and Fig 1D PANC-1 4E-BP1 in [2]
  - Fig 1C HT29 4E-BP1 in [1] and Fig 1D CFPAC-1 4E-BP1 in [2]
  - Partial or full panel similarity between Fig 1C SW948 4E-BP1 in [1], Fig 1C DLD-1 4E-BP1 in [1], Fig 3A right panel 4E-BP1 in [1], Fig 1D Mia-Paca2 4E-BP1 in [2] and Fig 1D Capan-1 4E-BP1 in [2].
- The Fig 1A β-actin panel in this article [1] appears similar to the Fig 1A β-actin panel in [3] when flipped horizontally.

Co-corresponding author CH stated that the underlying data for this article [1] are no longer available. In light of the above concerns that question the reliability of the results and which cannot be resolved in the absence of the underlying data, the *PLOS One* Editors retract this article.

CH agreed with the retraction and stands by the article’s findings. QW, FW, CL, GL, GW, TL, and ACB either did not respond directly or could not be reached.

The *PLOS One* Editors noted that the Statistical analysis subsection of the Materials and Methods section reports that data were analyzed using Student’s *t*-test; however, multiple experiments in this article [1] appear to present more than two variables. Additionally, the Materials and Methods section does not describe whether humane endpoints were applied in the animal studies, and the top of the Control error bar at 21 days in Fig 5A appears to have been cut off. Due to the above unresolved image concerns, the *PLOS One* Editors did not follow up on the methodological concerns further.
